# The Scottish Retinal Detachment Study: 10-year outcomes after retinal detachment repair

**DOI:** 10.1038/s41433-025-03613-8

**Published:** 2025-01-24

**Authors:** James E. Hazelwood, Danny Mitry, Jaswinder Singh, Harry G. B. Bennett, Ashraf A. Khan, Colin R. Goudie

**Affiliations:** 1 Princess Alexandra Eye Pavilion, Chalmers St, Edinburgh, EH3 9HA UK; 2 The University of Edinburgh, Edinburgh, UK; 3 Royal Free London, Pond St, Hampstead, London, NW3 2 QG UK

**Keywords:** Retinal diseases, Outcomes research

## Abstract

**Objective:**

To address the paucity of long-term data on outcomes following rhegmatogenous retinal detachment (RRD) repair we aimed to establish the 10-year best corrected visual acuity (BCVA), redetachment rate and lens status for patients from the Scottish Retinal Detachment Study.

**Subjects:**

Data from patients who presented with RRD during the original study were collected from clinical records 10 years after repair. Patients were excluded if lacking 10 year follow-up data, and excluded from visual acuity analysis in the case of significant co-morbid ophthalmic disease.

**Results:**

103 patients had BCVA outcomes for at least 10 years post-operatively and met the inclusion criteria. 57 were macula-on and 46 were macula-off. Median10-year BCVA was 0.1 (IQR 0.3) logMAR (6/7.5). 10-year BCVA was significantly better in macula-on patients, compared to macula-off (−0.18 logMAR (*p* < 0.001)). There was a significant improvement in macula-off BCVA from short-term follow-up to 10-year BCVA (−0.26 logMAR, *p* = 0.04). 93% of macula-on patients achieved BCVA sufficient for UK driving standard compared to 65% of macula-off. There was no difference in 10-year BCVA between repair techniques. Thirty-four patients were phakic at follow-up, 65 pseudophakic, and 4 aphakic. Redetachment occurred in 14% and conferred a poorer 10-year BCVA (logMAR 0.3 IQR 0.78 (6/12)).

**Conclusion:**

Long-term BCVA remains excellent following successful macula-on RRD repair with almost all macula-on, and most macula-off patients achieving the UK visual acuity driving standard. We demonstrate that macula-on detachments have significantly greater long long-term visual acuity than macula-off detachments, and that re-detachment is uncommon but confers a poorer long term visual outcome. This study provides objective long-term data to guide patient and surgeon expectations following retinal detachment repair.

## Introduction

Rhegmatogenous retinal detachment (RRD) affects approximately 12 in 100,000 people and is the most common ophthalmic emergency in the United Kingdom [1–3]. The separation of neurosensory retina from the pigment epithelium necessitates prompt intervention to prevent vision loss, with pars plana vitrectomy (PPV), scleral buckling (SB) and pneumatic retinopexy being the most common interventions. All three procedures achieve relatively high anatomical success rates of around 80-90% in uncomplicated cases [3–6].

Functional and visual outcomes can be more difficult to predict. “Macula-off” detachments are known to lead to poorer visual outcomes compared to “macula-on” detachments, and it is generally accepted that the duration of macular detachment prior to repair is an important prognostic factor in the final visual outcome. However, there is a lack of long term, prospective data to describe outcomes following retinal detachment repair, with most existing studies quoting visual acuity outcomes, usually from 3–12 months following repair [6–10]. Following successful repair, patients are commonly discharged from hospital follow-up within a few months and so long-term follow-up data is not routinely collected. Improvement in visual function following macular surgery can be slow, and as such optimal visual function following macula off RRD repair may not have been reached within this timeframe and maximal improvement not captured [11–13].

Furthermore, cataract development is significantly accelerated by vitrectomy and as part of the consent process patients should be informed that they will require cataract surgery sooner than if they were not to have surgery, particularly if aged over 50.

The Scottish Retinal Detachment Study was a prospective population-based study that recruited 96% of incident cases of RRD in Scotland over a two-year period [14]. Data collection was completed 10 years ago, providing a large, inclusive patient cohort from which population-level inferences can be drawn. This study described important demographic and clinical information, including the incidence, risk factors and clinical features of RRD, anatomical success rate, risk factors for failure and visual acuity after 1 year [15–17].

The aim of the present study was to establish long-term outcomes, including visual acuity, the number of patients suffering a re-detachment and the need for cataract surgery in patients who were recruited into the Scottish Retinal Detachment Study in one centre (Edinburgh), 10 years after their initial repair.

## Methods

The Scottish Retinal Detachment Study recruited 1244 patients from the 6 vitreoretinal surgical sites in Scotland (population of 5,168,500 in 2008) between 1st November 2007 and 21st October 2009. The methodology for the original Scottish Retinal Detachment Study is described in detail elsewhere [14]. The present study collected clinical information for all patients operated on in NHS Lothian (population circa 1,000,000) 10 years after initial retinal detachment. Data collected included best-corrected visual acuity (VA), lens status, ocular co-morbidities and redetachment rate. Data were collected from paper and electronic clinical records and when long-term visual acuity was unavailable, patients were contacted and asked to attend their optometrist for an up-to-date examination and measurement of visual acuity. If the patient was unable or unwilling to attend their optometrist, they were invited to attend the hospital clinic (Princess Alexandra Eye Pavilion) for assessment. Informed consent was obtained, and research adhered to the Declaration of Helsinki.

In cases of profound low vision or near blindness, perception of counting fingers, hand movements, light perception (LP), and no LP were substituted by LogMAR values of 2.0, 2.3, 2.8, and 3.0, respectively. Number of days from macular detachment to repair was based on patient reported date of central visual loss, collected in the original study.

Patients with less than 10 years of follow-up data were excluded from the study. Patients were excluded from visual acuity analysis if they had an alternative cause for reduced visual acuity (surgical glaucoma, previous retinal detachment or redetachment, age-related macular degeneration), but were eligible for re-detachment analysis. When analysing the relationship between number of days since macular detachment and 10 year BCVA patients reporting more than 2 weeks of central visual loss were excluded.

Descriptive statistical analysis was performed on the above variables using R (Version 4.3.2). The differences between macula-on and -off 10-year BCVA outcomes were assessed using the Wilcoxon rank sum test, similarly for comparing 10-year BCVA for those having a vitrectomy to those not. Wilcoxon signed rank was used to compare short-term (6 months) and long term 10-year BCVA outcomes. Spearman’s Rank was used to assess correlation between days of central visual loss and 10-year BCVA, with logistical regression used to explore this trend. Redetachment rates by repair technique were explored using Fisher’s exact tests.

This study was approved by Scotland A Research Ethics Committee (MREC-06/MRE00/19).

## Results

Two-hundred and fifty nine of the 1244 patient in the original study were repaired in Lothian and therefore eligible for our study. 129 patients had 10-year BCVA outcomes, with 103 meeting the inclusion criteria for acuity analysis. 46 patients were macula-off at presentation, 57 macular-on, 55 male, 48 female.

**Fig. 1:**
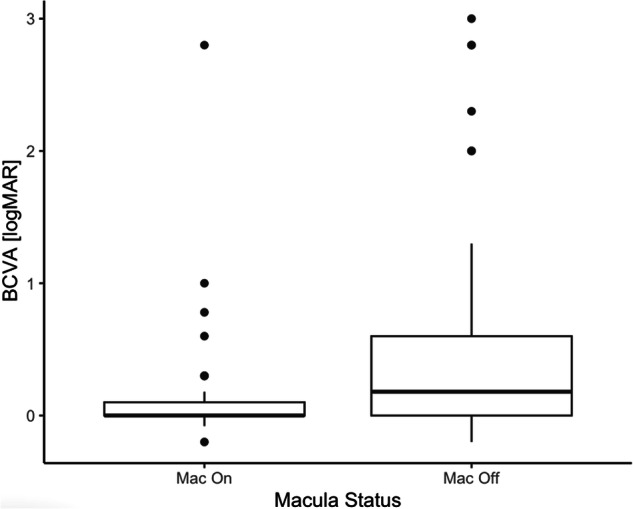
10-year BCVA outcomes for patients categorised by macular status at presentation.

Median post-operative 10-year BCVA was 0.1(IQR 0.3) logMAR (6/7.5). Median macula-on 10-year BCVA was 0.0 (IQR 0.1) logMAR (6/7.5) and median macula-off 10-year BCVA was 0.18 (IQR 0.6) logMAR, a significant difference of −0.18 (*p* < 0.001) logMAR (Fig. 1).

Median BCVA at short term follow-up (6 months) was 0.48 (IQR 0.42). There was a significant difference between short- and long-term VA for all patients (−0.28 *p* = 0.03) and for macula-off (−0.26, *p* = 0.04) with insufficient data for macula-on detachments due to original study design.

34 patients were phakic at follow-up (87 pre-operatively), 65 were pseudophakic (13 pre-operatively) and 4 aphakic (3 pre-operatively). Median BCVA at 10 years of phakic patients was 0.0 (IQR 0.24) (6/6), and for those pseudophakic 0.0 (IQR 0.3) (6/6). Of the 41 who had a vitrectomy, only 4 remained phakic.

Regarding repair technique performed, 34 patients had a vitrectomy, 25 had a scleral buckle, 7 had combined vitrectomy-buckle, 1 had a pneumatic retinopexy and 36 were not described. There was no difference in 10-year BCVA when comparing repair technique for all patients, or when analysed by subset of either macular-on or macular-off detachments.

### Macula-off

10-year BCVA data was available for 46 patients who presented with macula-off retinal detachment. Median 10-year BCVA was 0.18 (IQR 0.6) with 65.2% (*n* = 30) achieving 6/12 and 69.6% (*n* = 32) achieving 6/18. There was a significant difference between BCVA at 6 months, and BCVA at 10 years (−0.26 logMAR, *p* = 0.04) in this macula-off group. 25 had a vitrectomy, 16 had a scleral buckle and 5 had a combined vitrectomy buckle (10 unrecorded). There was no significant difference in 10-year BCVA between those having a vitrectomy or not (*p* = 0.41). At long term follow-up, 29 of 46 of macula off patients were pseudophakic (8 pre-op), 15 phakic (36 pre-op) and 2 aphakic (1 pre-op). Of the phakic patients, only 3 had undergone a vitrectomy.

**Fig. 2:**
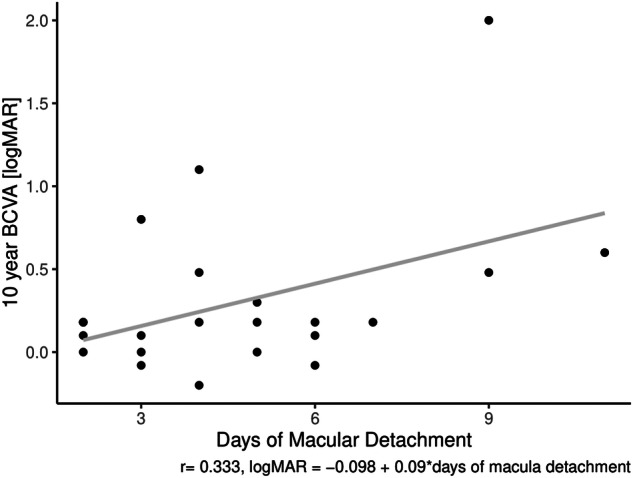
The relationship between days of central visual loss prior to surgery, and 10-year BCVA.

As the number of days since central vision loss until repair increased, 10-year BCVA decreased (Fig. 2). However, this relationship was not statistically significant (*r* = 0.333, *p* = 0.13).

### Macula-on

10-year BCVA was available for 57 patients who presented with macula-on retinal detachment. Median macula-on BCVA was 0.0 (IQR 0.1) logMAR (6/6), with 93% achieving >6/12 (*n* = 53). 16 patients had a vitrectomy, 16 had a buckle and 2 had vitrectomy-buckle. There was no significant difference in long-term VA between those who had a vitrectomy and those who did not (*p* = 0.50). At long term follow-up, 36 of 57 of macula on patients were pseudophakic (5 pre-op), 19 phakic (50 pre-op) and 2 aphakic (2 pre-op). Of the phakic patients, only 1 had had a vitrectomy.

### Redetachment

Retinal redetachment occurred in 18 out of 129 patients (14%) within 10 years (2 vitrectomy, 6 scleral buckle, 1 combined vitrectomy-buckle, 2 pneumatic retinopexies and 7 unrecorded). Patients were more likely to redetach if they had not had a vitrectomy (*p* = 0.048) (8/36 non-vitrectomy, 3/45 vitrectomy). This was also the case for the macula-on patients (*p* = 0.027) (6/22 Non-vitrectomy, 0/17 vitrectomy), but not in the macula-off cohort (*p* = 0.63). The median 10-year BCVA of patients with redetachments was poorer than those who did not suffer redetachment (0.3 logMAR, IQR 0.78), though that more of the redetaching patients were initially macular-off may confound this observation.

## Discussion

This study presents long-term 10-year visual acuity and clinical outcome data for patients following retinal detachment repair. Results demonstrate, as expected, that patients presenting with a macula-on retinal detachment have an excellent prognosis. Macula-off patients had a poorer 10-year BCVA, with a significant negative relationship between duration of macular detachment and BCVA at 10 years. However, most meet the UK driving acuity standard after 10 years and BCVA continues to improve past initial follow-up. Redetachment was uncommon and conferred a poorer 10-year BCVA.

In our cohort of macula-on retinal detachment patients, the mean 10 year BCVA of 0.11 logMAR compares favourably to the Primary Retinal Detachment Outcomes (PRO) study, which reports postoperative mean visual acuity for macula on patients of 0.22 LogMAR (20/33) in both phakic and pseudophakic patients (mean follow-up 399 and 388 days respectively) [9, 10]. Difference in results may in part be explained by the limited follow-up of little more than 1 year in the PRO study, given our results show BCVA to continue to improve past this timeframe, albeit in macula-off patients.

In macula-off patients, we demonstrate that BCVA continues to significantly improve years after repair, and that 65.2% achieved BCVA sufficient for UK driving standard (<0.3 logMAR), and 69.6% achieved 6/18 (<0.4 logMAR). Again, this compares favourably to reference data, with RCOphth NOD audit describing 46.3% achieving 0.3 logMAR (6/12) at 6 months, and the Scottish Retinal Detachment Study reporting 65.9% at 0.4 logMAR (6/15) at 1 year [3, 5]. Although some of the differences in our study may be explained by subsequent cataract surgery, both our data and previous work show continued improvement in BCVA after more than 1 year. The mechanism for this improvement is described by Fig. using en-face OCT analysis of the ellipsoid zone. They demonstrate that improvement in both retinal structure can continue for years after retinal detachment repair, and is associated with improved function [18].

Due to the emergent nature of retinal detachment repair, there is a lack of prospective evidence regarding the relationship between duration of macular detachment and reduction in long-term visual acuity. The Royal College of Ophthalmologists, under guidance from the British and Eire Association of Vitreoretinal Surgeons, suggests that macula-off detachments should be repaired as soon as possible, preferably within 3 days [3, 19]. In practice, evidence suggesting that post-operative visual acuity decreases the longer the macula remains detached is weighed against limited resource (manifest by a large variety in theatre capacity and availability of vitreoretinal specialists between different regions) throughout the UK and Ireland [20, 21]. Yorston et al. show that duration of macular detachment is associated with poorer outcomes. This agrees with our work with a trend to significance in the relationship between BCVA at 10 years and duration of macular detachment. However, our data show no significant difference in BCVA between those <3 d and >3 d of macular detachment in comparison to their results which recommend 72 h as a cut-off for repair [19].

A redetachment rate of 14% is similar to previous published data [3, 6–8]. Patients were more likely to redetach if they had not had a vitrectomy, though generalisation of this finding is limited due to the smaller sample size of the redetaching cohort. Ten-year BCVA was poorer in those who had a redetachment, but this is likely at least partly due to a greater proportion of the redetachments being initially macula-off.

The development of cataract is significantly accelerated by vitrectomy. Our results show only 9.8% remained phakic 10 years after vitrectomy, similar to that of Yee et al. who found 87% of patients had cataract surgery within 24 months following extensive vitrectomy for vitreous opacities similar to 80% of patients 29 months after vitrectomy for ERM [22, 23]. The likely need for subsequent cataract surgery is an important part of the consent process for retinal detachment repair with vitrectomy, especially if the patient is aged over 50. It was therefore interesting to note that 4 of 41 patients initially treated with vitrectomy remained phakic long-term, though degree of cataract was not assessed. Two had BCVA > 6/9, but 2 had counting fingers or worse though with a history of amblyopia.

The main limitation of this study is the attrition bias inherent in any long-term follow-up study. To maximise the sample size, we used the most recent BCVA recorded in hospital or by an optometrist. We collected 10-year outcome data for around half of all retinal detachment patients originally included in the study, which we feel is both satisfactory and expected, especially in light of patient migration, use of different primary eye care providers and lack of long-term follow-up in the hospital eye service. The use of ‘duration of central vision loss’ as a proxy to ‘days of macula detachment’ should also be considered. These are not necessarily equivalent as some patients remain ‘asymptomatic’ despite macular detachment, and relying on patient reported data reduces data precision and introduces recall bias [24]. However, this issue is not unique to our study and is one of the reasons that high quality data in this area lacking.

## Conclusion

This study provides the first long-term 10-year outcome data following retinal detachment repair. We demonstrate that BCVA is likely to remain excellent 10 years after successful macula-on RRD repair. Further, around two thirds of macula-off patients will achieve BCVA sufficient to meet the UK driving standard. Importantly, we have demonstrated that vision continues to improve past the normal follow-up interval after repair. Most patients are pseudophakic at follow-up, though a small number remain phakic. Redetachment is uncommon but confers a poorer visual outcome and in this cohort was more common in those without vitrectomy. This study provides important and objective data regarding expected outcomes after retinal detachment repair and can be used to guide clinician and patient expectations.

## Summary

### What was known before

- Rhegmatogenous retinal detachment (RRD) is the most common ophthalmic emergency and requires urgent repair to prevent irreversible visual loss.
- Pre-operative macular status is the major prognostic factor for visual acuity outcomes following retinal detachment repair.

### What this study adds

- Long-term visual acuity remains good in almost all patients following successful macula-on retinal detachment repair, and the majority of macula-off retinal detachment patients will eventually meet UK driving acuity standard, with vision continuing to improve even years after repair.
- In macula-off detachments, visual acuity continues to improve after initial short-term follow-up period.

## Data Availability

Datasets analysed in this study are available on reasonable request.
